# Temperament Trait Changes in Japanese Black Cows Under Grazing and Confined Conditions

**DOI:** 10.3389/fvets.2021.705764

**Published:** 2021-09-10

**Authors:** Noriaki Nakajima, Hiroki Mitsuishi, Masato Yayota

**Affiliations:** ^1^The United Graduate School of Agricultural Science, Gifu University, Gifu, Japan; ^2^Faculty of Applied Biological Sciences, Gifu University, Gifu, Japan; ^3^Education and Research Center for Food Animal Health, Gifu University, Gifu, Japan

**Keywords:** confinement, docility, grazing, temperament trait, visual analog scale

## Abstract

The objective of the present study was to reveal the effects of grazing on the temperament traits of cows. Nine Japanese Black cows [344 ± 32 kg body weight (BW), 7.7 ± 3.0 year of age], which had various experiences, such as tethering, handling, and grazing, were used in this experiment. Five of the nine cows were grazed for 3 months on a 1.8-ha field composed of a sown pasture with forestland. The remaining cows were fed in confinement. On days 38, 52, 72, and 86 after the start of grazing, the temperament traits observed in various situations, such as moving to the body weight scale, weighing, handling, moving to the stock for blood sampling, holding in the stock, and obtaining a blood sample, were assessed with a visual analog scale (VAS: 1–10) or score (1–5). During weighing and handling, the intensity of resistance exhibited by the grazing cows, as evaluated by head movement, walking/stepping, tail flicking, rope tension, and overall movement, was lower than that exhibited by confined cows (*P* < 0.05). The resistance score exhibited by the grazing cows during blood sampling was also lower than that exhibited by confined cows (*P* < 0.01). These results suggest that grazing enhances docility in cows with various experiences in different situations encountered in daily management.

## Introduction

In cattle, temperament is described as an animal's response to handling or forced movement by humans (1). Farmers use the term “temperament” to describe cattle behavior during handling. Temperament is one of the most important parameters in livestock production. It contributes to animal productivity and meat quality (2), animal welfare (3, 4), immunity (5, 6), and even the safety of those handling the animals (7). For example, cattle with excitable temperaments have lower average daily gain and higher mortality rates than those with calm temperaments (8, 9), suggesting that the temperament of cattle is a critical parameter for farmers because of the monetary impact.

Handling and rearing can affect cattle temperament. Cattle that are frequently handled tend to become more docile than those that are less handled (10); however, excessive handling could be detrimental in animal management with regard to human safety since these individuals develop no flight zone. In contrast, extensively managed beef cattle are relatively unfamiliar with humans (11). Less frequent human-animal interactions make cattle fearful, which can cause them to behave aggressively during handling (10). Social interactions between animals also affect their temperament (12). The presence of peers reduces the stress responses to fear-inducing situations in cattle (13). When peers are in sight, heifers display less behavior indicative of distress in response to a novel object (14). Cows engage in many more active social interactions with other individuals when they are grazed than when they are confined with tethering (15); thus, grazing could have a positive impact on temperament *via* social interactions.

Physical condition also relates to temperament. Calm and excitable cattle have different cortisol concentrations (16). Grazed cows have lower cortisol concentrations than confined cows (17). The change in stress susceptibility could change the behavior of cattle during handling and restraint. However, a report showed that calves kept indoors were calmer than calves kept outdoors (18), implying that grazing may adversely affect temperament. This inconsistency in the relationship between grazing and temperament traits may be attributable to the frequency of contact between humans and cattle (10). In addition, cattle's previous experiences can shape their future reactions to humans (10). For example, a negative experience such as poor handling and holding in a yard environment by the handler increases cattle reactivity (19), whereas a positive experience such as gentle handling by the handler reduces animal reactivity in future handling (20). Generally, the system used to rear Japanese Black beef cows requires frequent contact with humans during daily management practices. Japanese Black beef cows gain experience with handling, tethering and other types of interactions during the rearing process. Thus, the effects of grazing on the temperament of calves may not be the same as the effects of grazing on the temperament of adult dairy cows and beef cows that come into contact with humans on a daily basis. The visual analog scale (VAS) is a quantitative assessment with high intra- and inter-observer reliability. It is considered a reliable and practical assessment method for cattle temperament evaluation, although it is not generally superior to other methods (21).

The aim of this study was to reveal, mainly using the VAS, whether grazing affects the temperament of beef cows in various situations encountered in daily management practices, such as weighing, handling, and blood sampling.

## Materials and Methods

### Animals, Housing, Grazing, and Diets

This study was conducted at the Minokamo livestock farm, Gifu Field Science Center, Gifu University (longitude 137°03′57″E; latitude 35°26′44″N), from June to August 2018. Nine Japanese Black cows (344 ± 32 kg body weight (BW), 7.7 ± 3.0 year, not lactating and not pregnant) with no clinical signs of disease and no external injury at the start of the experiment were used. All cows were housed in an 8 m × 7.3 m indoor pen and tethered to tie stalls in a closed barn for the first 2 weeks of the experiment. Each cow was tethered with a rope but was able to engage in social interactions with neighboring individuals. The pen had a concrete floor covered with sawdust bedding. The cows were fed ~5 kg/day Sudan grass hay, 1 kg/day wheat bran and 50 g/day calcium phosphate on an as-fed basis at 08:00 and 16:00 h according to the Japanese Feeding Standard for beef cattle (22). The cows had free access to water and mineral salt blocks. Then, five of the nine cows were rotated as a group between grazing on a 1.8-ha pasture composed of sown pasture, which was dominated by Italian rye grass [*Lolium multiflorum* (Lam.)], and a forestland for 3 months (**grazed cow: GC**). The dry matter (DM) content and grass height of the herbage in the sown grassland were 22.4% and 62.9 cm, respectively. The grazing area was divided into four paddocks, and the cows were rotated among the paddocks based on the availability of forage. The grazing cows remained outside all day and consumed only the herbage in the pasture, with access to a mineral salt block and water. The remaining cows were maintained under the confined conditions described above (**confined cow: CC**) for 3 months. The cows were allocated to the conditions so that average weight and age were matched as closely as possible between groups. In addition, all cows were old enough to have considerable experience with tethering, handling by humans and grazing in farm management. The frequency of daily monitoring was the same for the GCs and CCs, although the CCs also came into contact with farm staff when they were fed and when their pen was cleaned. The mean ambient temperature and humidity were 27.2 ± 5.1°C and 69.7 ± 19% during the experiment, respectively.

### Temperament Trait Analysis

**Figure 1:**
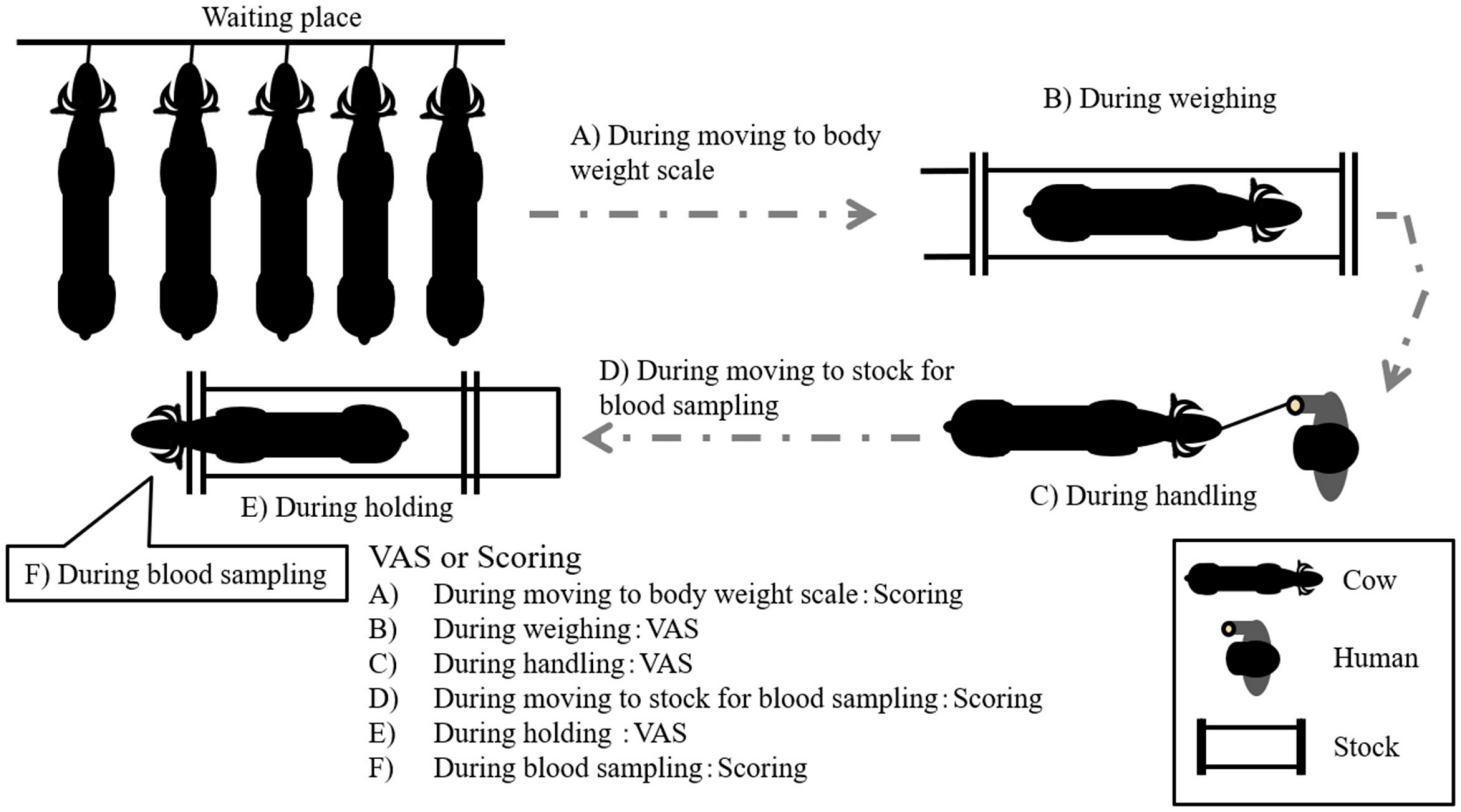
Outline of the evaluation of temperament.

The average BW and age of GCs were 351 ± 30 kg and 10.0 ± 2.1 year, respectively, whereas those of CCs were 336 ± 37 kg and 9.8 ± 4.2 year, respectively. The age of all cows was over 6 year. The temperament traits of the cows were observed on days 0, 38, 52, 72, and 86 after the start of grazing. The recording of the temperament traits started at 08:00 h before feeding. The observational procedure was as follows: First, a handler moved each cow from a waiting place to a body weight scale using a handling rope (Figure 1A). The distance from the waiting place to the body weight scale was ~15 m. The intensity of cow resistance during this movement was recorded and analyzed using the scoring system described below (**Table 2**). Then, the cows were weighed on the scale, and the intensity of cow resistance with regard to each individual behavior was recorded for 2 min [visual analog scale (VAS): 21] (Figure 1B). After weighing, the cow was held in one place by the handler for 2 min (Figure 1C). The distance from the body weight scale to the location for holding by the handler was ~2 m. The length of the rope from the cow to the handler was kept at ~1 m. The intensity of the cow's resistance with regard to each behavior while standing was recorded and analyzed with the VAS. Subsequently, the handler moved the cow into a stock to enable a blood sample to be drawn (Figure 1D). The distance from the location for holding by the handler to the stock used for blood sampling was ~30 m. The intensity of the cow's resistance while moving to the stock was recorded and analyzed using the scoring system (**Table 2**). In the stock, the cow's behaviors were recorded for the first 2 min and analyzed with the VAS (Figure 1E). Finally, the intensity of the cow's resistance during blood sampling was recorded and analyzed using the scoring system (**Table 2**). The collected blood samples were used for further analysis (section Statistical analysis). During the behavioral test, the times of the behavioral test and waiting time per cow were ~30 min and an hour and a half, respectively, per behavioral test.

The behaviors of all cows were recorded using two video cameras (GZ-MG575, Victor Co., Ltd., Yokohama, Japan) and were analyzed using a VAS (21) or scoring system (Figure 1). The VAS is a continuous horizontal scale. This assessment is used to measure the intensity of a behavior on a ten-centimeter scale in analog format (Table 1). The behaviors assessed with the VAS were overall movement, head movement, tail flicking, walking/stepping, and tension of the handling rope, as shown in Table 1. The inter- and intra-observer reliability of the VAS was confirmed by Vogt et al. (21). The intensity of resistance during the handling procedure was recorded using a scoring system (Table 2). This scoring system classified the degree of resistance into five stages from “no resistance [1]” to “intense resistance [5].” Scoring was conducted using a video clip to minimize scoring differences between observers. All video clips were analyzed by two observers. Observers were blinded as to which individual was in grazing or confinement when the behaviors of cows were analyzed by the VAS. The correlation between observers with regard to the VAS scores is also shown in Table 3.

**Table 1:** Definition of temperament traits and visual analog scale (VAS).

	**Endpoints of VAS**		**Definition**
**Temperament trait**	**Min (0)**	**Max (10)**	
Head movement	No movement	Head permanently moving/violent struggling	The head is displaced horizontally and/or vertically in relation to the median plane (23)
Tail flicking	No flicking	Constant flicking	Tail movement to the left or right of the center and back again, i.e., a tail movement from the left to the right side would count as two flicks (24)
Walking/stepping	No walking/stepping	Continuous walking/stepping	Two or more limbs are alternately raised and make contact with the ground again, with or without ground covered between movements
Rope tension^a^	Loose	Tightened	Evaluates whether the rope used to tie the cattle forms a curve (relaxed) or a straight line (tensed) (25)
Overall movement	calmness	Wild/Aggressive	

a *The tethering test only*.

**Table 2:** Definition of temperament trait score.

**Timing**	**Temperament trait score**				
	**1**	**2**	**3**	**4**	**5**
During moving to body weight measurement scale	No resistance	Almost no resistance	A handler approaches and chases the individual from behind the cow	A handler pushes the individual hard from behind the cow “or” pulls a handling rope strongly from the front of the cow	A handler pushes the individual hard from behind the cow “and” pulls a handling rope strongly from the front of the cow
During moving to stock for blood sampling					
During blood sampling	No resistance	Slight resistance	Moderate resistance	Considerable resistance	Extreme resistance

**Table 3:** Correlation (*r*) of visual analog scale (VAS) between two observers.

**Temperament trait**	**Correlation (** ***r*** **)**		
	**Weighing**	**Tethering**	**Holding for blood sampling**
Overall movement	0.86	0.79	0.74
Head movement	0.89	0.76	0.64
Tail flicking	0.75	0.72	0.53
Walking/stepping	0.88	0.89	0.71
Rope tension^a^	–	0.83	–

a *The tethering test only*.

### Blood Analysis

Blood samples were collected from the jugular vein using a vacuum collection tube containing heparin (Venoject II vacuum blood collection tube, TERUMO Co., Ltd., Tokyo, Japan). Blood samples were centrifuged at 1,000 × g at 4°C for 10 min to collect the blood plasma. The plasma samples were stored at −80°C until cortisol analysis. The concentration of cortisol was determined using a commercial kit (Cortisol EIA Kit, Oxford Biomedical Research, Inc., MI, USA).

### Statistical Analysis

We calculated the sample size using G^*^Power version 3.1.9.2 (two-way ANOVA with repeated measures, α = 0.05, (1–β) = 0.8, University of Dusseldorf, Dusseldorf, Germany). Power analyses of temperament traits and blood parameters showed that appropriate power (0.8 or above) to detect differences in 11 of the 17 parameters could be obtained with a total sample size of nine or fewer animals. Considering the cost and availability of experimental cows, the sample size was determined based on the assumption of large effect sizes. The statistical unit in this experiment was the individual animal rather than the treatment group. This unit was chosen because the grazing period lasted 3 months, and it would have been difficult to create several replicates of the grazing treatment due to the limited pasture and herd sizes and the long study period. The adequacy of this approach was described by Connolly (26).

All data were analyzed statistically using the lmerTest package (27) in R software (version 3.0.2: R core team, 2013). Normality tests were conducted using the Shapiro-Wilk test before the analysis. Then, the data were analyzed using a generalized linear mixed model (GLMM) with repeated measurements according to the data distributions. The treatment (GC vs. CC), sampling day (days 0, 38, 52, 72, and 86) and their interaction were considered to be fixed effects, and an individual animal was considered to be a random effect. When the data matched normal distribution, we estimated degrees of freedom, *F*- and *p*-values using type III ANOVAs with Satterthwaite's approximation. While the data matched Poisson and binomial distribution, we used Type II Wald chisquare tests for calculating degree of freedom, Chi-square and *p*-values. Differences were considered significant at *P* < 0.05. Trends were identified at 0.05 < *P* < 0.1.

## Results

### Temperament Trait Analysis

**Figure 2:**
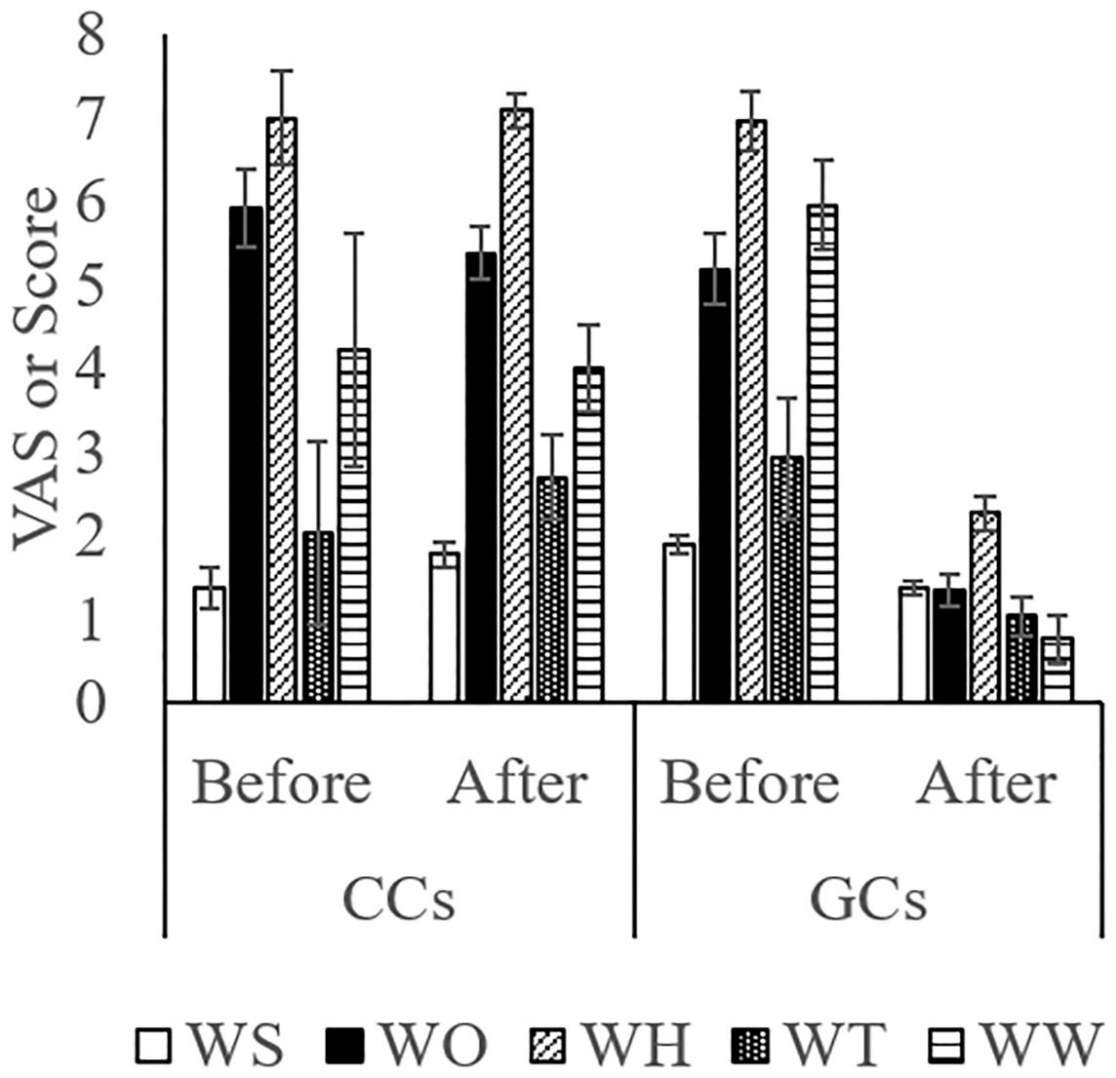
Temperament while moving to the body weight scale and during weighing in GCs and CCs. The horizontal axis shows the temperament before and after the start of grazing. Data are presented as the means ± SEM. WS, The scoring of resistance during movement to the body weight scale; WO, The VAS score for overall movement; WH, The visual analog scale (VAS) score for head movement; WT, The VAS score for tail flicking; WW, The VAS score for walking/stepping.

**Figure 3:**
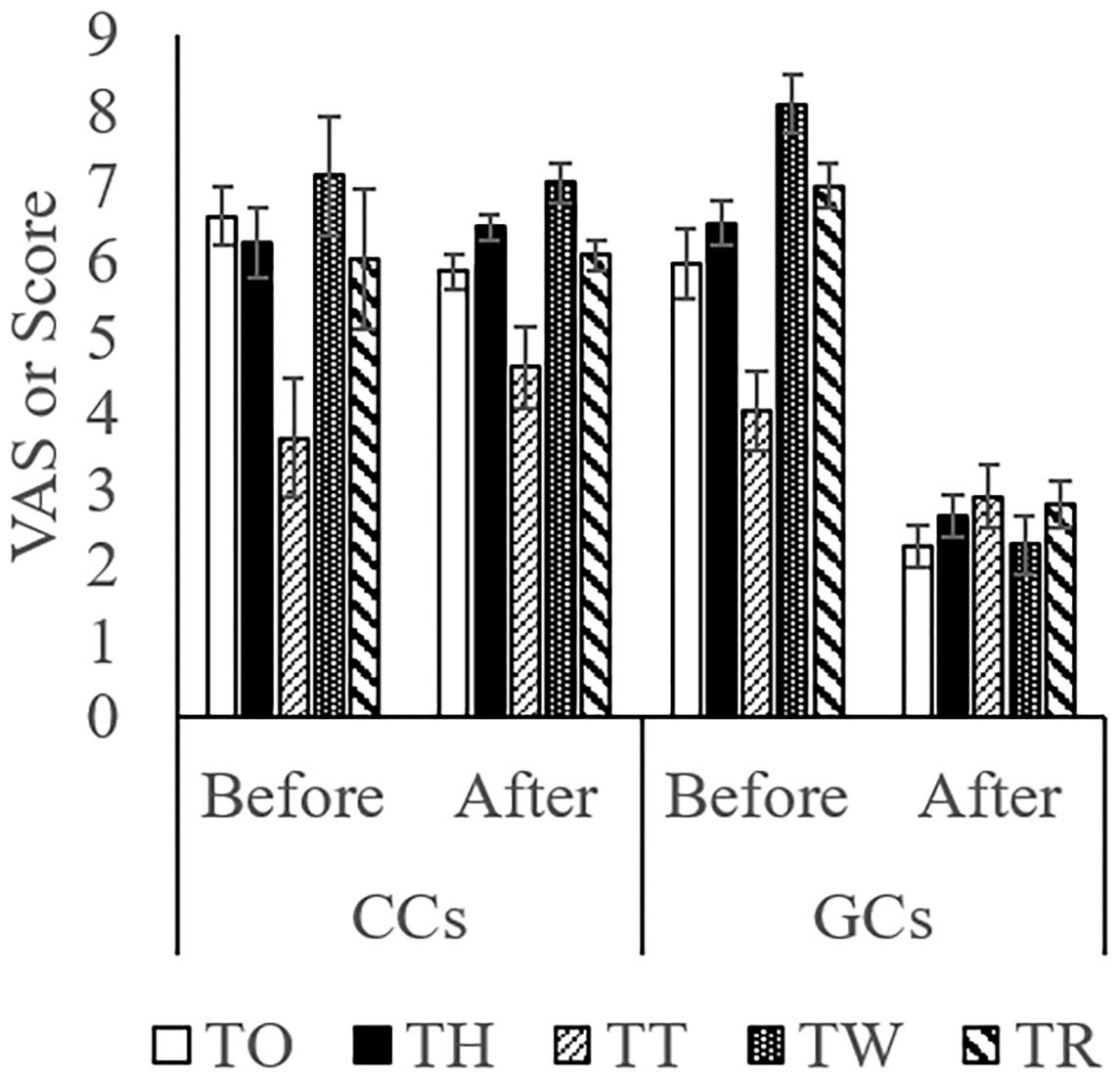
Temperament during handling in GCs and CCs. The horizontal axis shows the temperament before and after the start of grazing. Data are presented as the means ± SEM. TO, The VAS score for overall movement; TH, The visual analog scale (VAS) score for head movement; TT, The VAS score for tail flicking; TW, The VAS score for walking/stepping; TR, The VAS score for rope tension.

**Figure 4:**
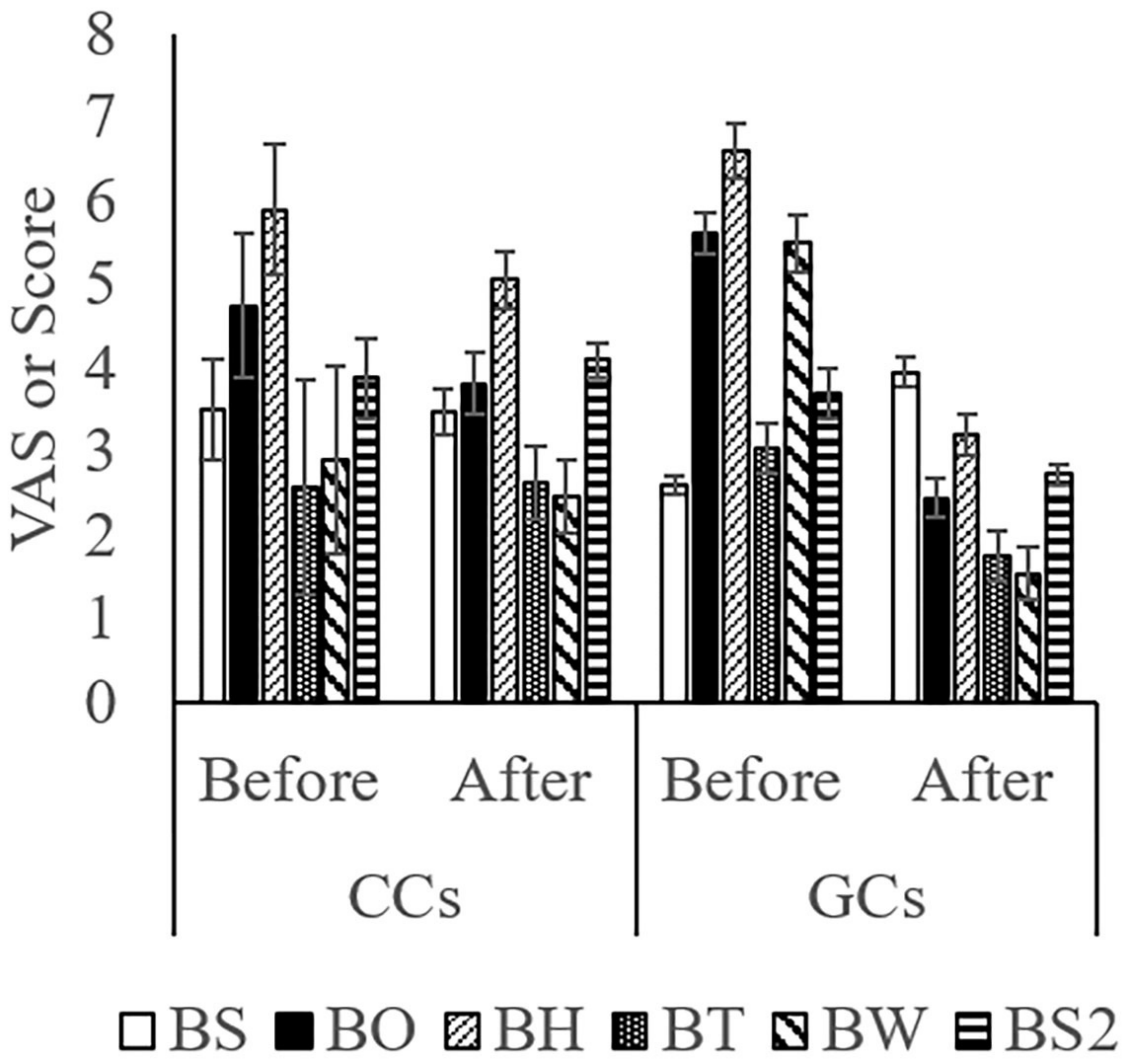
Temperament traits while moving to the stock for blood sampling, while in the device, and during blood sampling in GCs and CCs. The horizontal axis shows the temperament before and after the start of grazing. Data are presented as the means ± SEM. BS, The score for resistance while moving to the stock for blood sampling; BH, The visual analog scale (VAS) score for head movement; BT, The VAS score for tail flicking; BW, The VAS score for walking/stepping; BO, The VAS score for overall movement; BS2, The score for resistance during blood sampling.

The score while moving to the body weight scale (Figure 1A) was lower in GCs than in CCs after the start of grazing (*P* = 0.05: Figure 2; Supplementary Figure 1; Table 4). During weighing (Figure 1B), the VAS scores indicating resistance as expressed by overall movement, head movement, tail flicking, and walking/stepping were significantly lower in GCs than in CCs after the start of grazing (all behaviors: treatment: *P* < 0.01; Figure 2, Supplementary Figure 1, Table 4). During handling (Figure 1C), the VAS scores for resistance as expressed by head movement, tail flicking, walking/stepping, rope tension, and overall movement were significantly lower in GCs than in CCs after the start of grazing (all behaviors: treatment: *P* < 0.01; Figure 3; Supplementary Figure 2; Table 4). During movement to the stock for blood sampling (Figure 1D), there were no significant differences in resistance scores between GCs and CCs (treatment: *P* = 0.93). In the stock (Figure 1E), the VAS scores for overall movement (treatment: *P* = 0.09) and head movement (treatment: *P* = 0.02) were lower in GCs than in CCs (Figure 4; Supplementary Figure 3; Table 4). During blood sampling (Figure 1F), the resistance score was significantly lower in GCs than in CCs (treatment: *P* < 0.01: Figure 4; Supplementary Figure 3; Table 4).

**Table 4:** Statistical results of the temperament traits test of cows in each situation.

		**DF**			**Num DF**			**Den DF**			**F value or** **Chi-square value**			***P*** **-value**		
**Situation**	**Item**	**S**	**T**	**T × S**	**S**	**T**	**T × S**	**S**	**T**	**T × S**	**S**	**T**	**T × S**	**S**	**T**	**T × S**
During moving to the body weight scale and during the weighing	During moving to body weight scale	3	1	3	–	–	–	–	–	–	17.6^a^	3.8^a^	3.2^a^	<0.05	0.05	0.36
	Head movement	–	–	–	3	1	3	21	7	21	1.5	130.0	1.0	0.26	<0.01	0.42
	Tail flicking	3	1	3	–	–	–	–	–	–	1802.6^a^	9.2^a^	252.4^a^	<0.01	<0.01	<0.01
	Walking/Stepping	–	–	–	3	1	3	21	7	21	2.0	26.7	1.2	0.15	<0.01	0.35
	Overall movement	–	–	–	3	1	3	21	7	21	4.6	84.3	2.4	0.01	<0.01	0.1
During handling	Head movement	–	–	–	3	1	3	21	7	21	0.7	57.5	1.0	0.55	<0.01	0.39
	Tail flicking	–	–	–	3	1	3	21	7	21	18.3	6.6	0.8	<0.01	<0.01	<0.01
	Walking / Stepping	–	–	–	3	1	3	21	7	21	2.0	88.2	0.1	0.14	<0.01	0.96
	Rope tension	–	–	–	3	1	3	21	7	21	1.8	30.1	0.2	0.17	<0.01	0.89
	Overall movement	–	–	–	3	1	3	21	7	21	4.7	68.8	0.3	0.01	<0.01	0.8
During moving to the stock for blood sampling, holding in the device, and during blood sampling	During moving to stock	3	1	3	–	–	–	–	–	–	31.5^a^	0^a^	25.2^a^	<0.01	0.93	<0.01
	Head movement	–	–	–	3	1	3	21	7	21	2.3	8.5	2.4	0.1	0.02	0.1
	Tail flicking	–	–	–	3	1	3	21	7	21	4.1	1.9	2.7	0.02	0.22	0.07
	Walking / Stepping	–	–	–	3	1	3	21	7	21	0.5	1.7	2.0	0.71	0.23	0.14
	Overall movement	–	–	–	3	1	3	21	7	21	12.6	3.7	3.6	<0.01	0.09	0.03
	During blood sampling	3	1	3	–	–	–	–	–	–	3.6^a^	9.2^a^	18.5^a^	0.31	<0.01	<0.01

*DF, Degrees of freedom; Num DF, Numerator degrees of freedom; Den DF, Denominator degrees of freedom; T, Treatment: a significant difference between treatments (GC vs. CC); S, sampling day: a significant difference between sampling days (0, 38, 52, 72, and 86); T × S = treatment × sampling day interaction: a significant difference in the interaction of treatment × sampling day*.

a *Chi-square value. Differences were considered significant at P < 0.05. A tendency toward significance was indicated by 0.05 < P < 0.1*.

### Cortisol Analysis

**Figure 5:**
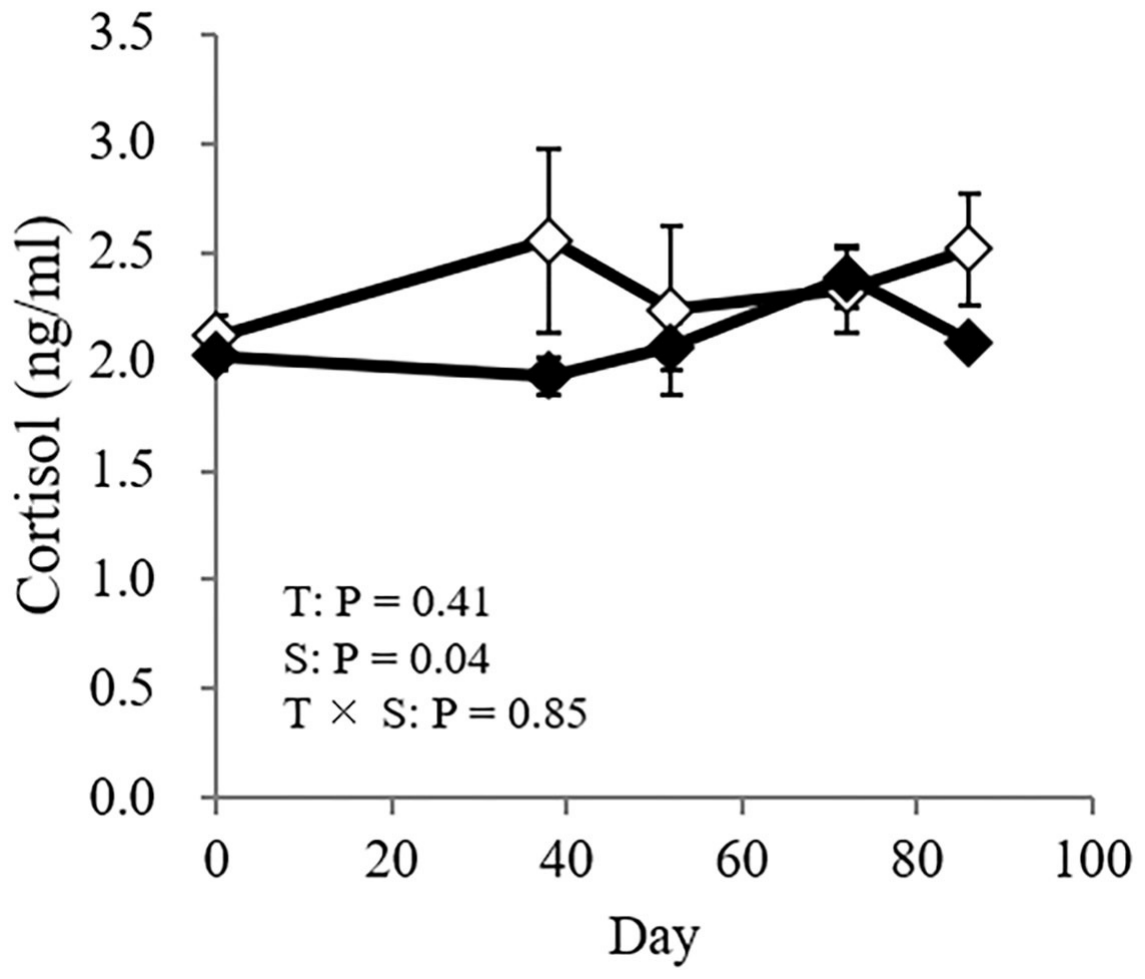
The cortisol concentration in GCs (♦) and CCs (♢). The horizontal axis shows the day on which the blood sample for the cortisol analysis was obtained, and day 0 (0) represents the day the cattle were moved to a pasture. Data are presented as the means ± SEM. T, Treatment: a significant difference between treatments (GC vs. CC); S, sampling day: a significant difference between sampling days (0, 38, 52, 72, and 86); T × S = treatment × sampling day interaction: a significant difference in the interaction of treatment × sampling day. Differences were considered significant at *P* < 0.05. A tendency toward significance was indicated by 0.05 < *P* < 0.1.

No significant difference was found in the concentration of cortisol between the treatments, nor was the interaction between treatment and sampling day significant (treatment: *P* = 0.41; interaction: *P* = 0.85: Figure 5).

## Discussion

The GCs were more docile than the CCs in various management situations, including weighing, handling, and holding for blood sampling. The GCs were also calmer while moving to the body weight scale and during blood sampling. These results suggest that grazing has strong impacts on the temperament of cows. The social environment is linked to temperament (12). Grignard et al. (13) reported that the existence of social partners improves the tractability of calves during handling (23). Moreover, housing conditions influence animal affective state and cognitive bias (28). Horses tend to judge optimistically in ambiguous situations when a positive affective state prevails by accessing pasture and contacting conspecifics (29). In general, animals were pessimistic when in a negative affective state, whereas they were optimistic when in a positive affective state. In the present study, GCs engaged in more active social interactions with other individuals than CCs, as we previously reported (15). Thus, although the presence of social partners during the temperament test, including handling and restriction, was the same in both treatments (grazing vs. confinement), the increase in social interaction between GCs and accessing pasture might be one of the factors reinforcing calmness during handling and restriction.

Stress susceptibility is another factor leading to more aggressive behavior. Aggressive individuals have high cortisol concentrations (30). In the present study, the cortisol concentrations in CCs were in the range of 1.6–3.8 ng/ml whereas that in GCs were in the range of 1.7–2.9 ng/ml after the start of grazing, and no significant difference was detected in cortisol concentrations between the GCs and CCs. The cortisol concentrations of both treatments in the present study were close to the basal value of previous studies (31, 32). Thus, the cows in the present study might not be under intense stress. However, Higashiyama et al. (17) reported that the concentration of urinary cortisol increased 3.4-fold when grazing cattle were moved to a confined space, whereas when confined cattle were moved to a pasture, the concentration of cortisol did not increase. Urinary cortisol showed a similar pattern to plasma cortisol with an ~0.5-h time lag (31). The increase in cortisol concentrations in those previous studies returned to baseline within hours to days, implying that it is necessary to evaluate the relative change of its concentration at short intervals. In addition, our previous study showed that grazing cows had higher antioxidant capacity than confined cows, implying that grazing cows are less susceptible to physiological stress (33). Thus, susceptibility to stress under different feeding conditions might still impact animal temperament, and further study is needed.

Experiences, including handling and transporting, affect temperament (34). Boivin et al. (18) reported that grazing caused a reduction in the expression of calm temperament traits in calves. The findings of the present study were inconsistent with the results of Boivin's study (18). However, the cows in the present study were adults that had experience with various management practices, including grazing, and were frequently handled by farm staff before this study. Moreover, age, breed, and genetics affect temperament (35). These traits of calves investigated in Boivin's study are different from those in the present study. Thus, the differences between the studies may induce different behavioral responses to grazing.

## Conclusions

The present study showed that grazing was related to higher docility of cows in various management situations, such as weighing, handling, and blood sampling. Grazing may have contributed to mitigating the reaction to human-cattle interaction during handling and the reactions of cattle to restraint and painful operations. This is the first study to suggest a relationship between grazing and temperament in cows. Further study is needed to reveal the relationships between temperament traits and environmental factors such as social connections (29), stress conditions or ingestion of plants with antioxidants (33) while grazing.

## Data Availability Statement

The original contributions presented in the study are included in the article/Supplementary Material, further inquiries can be directed to the corresponding author/s.

## Ethics Statement

All animal experimental procedures were approved by the Committee for Animal Research and Welfare of Gifu University (#17140) and conducted in accordance with the guidelines for animal research and welfare of Gifu University.

## Author Contributions

NN designed the study, collected the data, conducted the statistical analysis, interpreted the data, and wrote and developed the manuscript. MY contributed to designing the study and interpreting the data and reviewed and developed the manuscript. HM conducted most of the data sampling and experimental work. All authors contributed to the article and approved the submitted version.

## Funding

This research was funded by the United Graduate School of Agricultural Science, Gifu University. This research did not receive any specific grant from funding agencies in the public, commercial, or not-for-profit sectors.

## Conflict of Interest

The authors declare that the research was conducted in the absence of any commercial or financial relationships that could be construed as a potential conflict of interest.

## Publisher's Note

All claims expressed in this article are solely those of the authors and do not necessarily represent those of their affiliated organizations, or those of the publisher, the editors and the reviewers. Any product that may be evaluated in this article, or claim that may be made by its manufacturer, is not guaranteed or endorsed by the publisher.

## References

[1] Tulloh NM. Behaviour of cattle in yards. II. A study of temperament. Anim Behav. (1961) 9:25–30. 10.1016/0003-3472(61)90046-X

[2] Haskell MJ Rennie LJ Bowell VA Bell MJ Lawrence AB. Housing system, milk production, and zero-grazing effects on lameness and leg injury in dairy cows. J Dairy Sci. (2006) 89:4259–66. 10.3168/jds.S0022-0302(06)72472-9 17033013

[3] Borstel U. Assessing and influencing personality for improvement of animal welfare: a review of equine studies. CAB Rev. (2013) 8:1–27. 10.1079/PAVSNNR20138006

[4] Broughan C. Odours, emotions, and cognition-how odours may affect cognitive performance. Int J Aromather. (2002) 12:92–8. 10.1016/S0962-4562(02)00033-410461124

[5] Burdick NC Banta JP Neuendorff DA White JC Vann RC Laurenz JC. Interrelationships among growth, endocrine, immune, and temperament variables in neonatal Brahman calves. J Anim Sci. (2009) 87:3202–10. 10.2527/jas.2009-1931 19542503

[6] Fell LR Colditz IG Walker KH Watson DL. Associations between temperament, performance and immune function in cattle entering a commercial feedlot. Aust J Exp Agric. (1999) 39:795–802. 10.1071/EA99027

[7] Burrow HM. Measurements of temperament and their relationships with performance traits of beef cattle. Anim Breed Abstr. (1997) 65:477–95.

[8] Reinhardt CD Busby WD Corah LR. Relationship of various incoming cattle traits with feedlot performance and carcass traits. J Anim Sci. (2009) 87:3030–42. 10.2527/jas.2008-1293 19465501

[9] Voisinet BD Grandin T Tatum JD O'connor SF Struthers JJ. Feedlot cattle with calm temperaments have higher average daily gains than cattle with excitable temperaments. J Anim Sci. (1997) 75:892–6. 10.2527/1997.754892x 9110198

[10] Ceballos MC Góis KCR Sant'Anna AC da Costa MJP. Frequent handling of grazing beef cattle maintained under the rotational stocking method improves temperament over time. Anim Prod Sci. (2016) 58:307–13. 10.1071/AN16025

[11] Gauly M Mathiak H Erhardt G. Genetic background of behavioural and plasma cortisol response to repeated short-term separation and tethering of beef calves. J Anim Breed Genet. (2002) 119:379–84. 10.1046/j.1439-0388.2002.00360.x

[12] Nicol CJ. The social transmission of information and behaviour. Appl Anim Behav Sci. (1995) 44:79–98. 10.1016/0168-1591(95)00607-T

[13] Grignard L Boissy A Boivin X Garel JP Le Neindre P. The social environment influences the behavioural responses of beef cattle to handling. Appl Anim Behav Sci. (2000) 68:1–11. 10.1016/S0168-1591(00)00085-X 10771312

[14] Boissy A Le Neindre P. Social influences on the reactivity of heifers: implications for learning abilities in operant conditioning. Appl Anim Behav Sci. (1990) 25:149–65. 10.1016/0168-1591(90)90077-Q

[15] Nakajima N Doi K Tamiya S Yayota M. Physiological, immunological, and behavioral responses in cows housed under confinement conditions after grazing. Livest Sci. (2018) 218:44–9. 10.1016/j.livsci.2018.10.011

[16] Curley KO Jr Paschal JC Welsh TH Jr Randel RD. Exit velocity as a measure of cattle temperament is repeatable and associated with serum concentration of cortisol in Brahman bulls. J Anim Sci. (2006) 84:3100–3. 10.2527/jas.2006-055 17032804

[17] Higashiyama Y Nashiki M Narita H Kawasaki M. A brief report on effects of transfer from outdoor grazing to indoor tethering and back on urinary cortisol and behaviour in dairy cattle. Appl Anim Behav Sci. (2007) 102:119–23. 10.1016/j.applanim.2006.03.007

[18] Boivin X Le Neindre P Garel JP Chupin JM. Influence of breed and rearing management on cattle reactions during human handling. Appl Anim Behav Sci. (1994) 39:115–22. 10.1016/0168-1591(94)90131-7

[19] Petherick JC Doogan VJ Holroyd RG Olsson P Venus BK. Quality of handling and holding yard environment, and beef cattle temperament: 1. Relationships with flight speed and fear of humans. Appl Anim Behav Sci. (2009) 120:18–27. 10.1016/j.applanim.2009.05.008

[20] Becker BG Lobato JP. Effect of gentle handling on the reactivity of zebu crossed calves to humans. Appl Anim Behav Sci. (1997) 53:219–24. 10.1016/S0168-1591(96)01091-X

[21] Vogt A Aditia EL Schlechter I Schütze S Geburt K Gauly M von Borstel UK. Inter-and intra-observer reliability of different methods for recording temperament in beef and dairy calves. Appl Anim Behav Sci. (2017) 195:15–23. 10.1016/j.applanim.2017.06.008

[22] National Agriculture and Food Research Organization (NARO). Japanese Feeding Standard for Beef Cattle. Tokyo: Japan Livestock Industry Association (2008).

[23] Grignard L Boivin X Boissy A Le Neindre P. Do beef cattle react consistently to different handling situations? Appl Anim Behav Sci. (2001) 71:263–76. 10.1016/S0168-1591(00)00187-8 11248377

[24] Schwartzkopf-Genswein KS Stookey JM Welford R. Behavior of cattle during hot-iron and freeze branding and the effects on subsequent handling ease. J Anim Sci. (1997) 75:2064–72. 10.2527/1997.7582064x 9263052

[25] Boissy A Bouissou MF. Effects of early handling on heifers' subsequent reactivity to humans and to unfamiliar situations. Appl Anim Behav Sci. (1988) 20:259–73. 10.1016/0168-1591(88)90051-2

[26] Connolly J. Perspectives on the use of animal as replicate in grazing experiments. In: Sustainable Meat and Milk Production from Grasslands, Proceedings of the 27th General Meeting of the European Grassland Federation. Cork (2018). p. 404–6.

[27] Kuznetsova A Brockhoff PB Christensen RH. LmerTest: Tests for Random and Fixed Effects for Linear Mixed Effect Models. R package, version 2.0-11 (2014). Available online at: http://CRAN.R-project.org/package=lmerTest (accessed April 5, 2017).

[28] Harding EJ Paul ES Mendl M. Cognitive bias and affective state. Nature. (2004) 427:312. 10.1038/427312a 14737158

[29] Löckener S Reese S Erhard M Wöhr AC. Pasturing in herds after housing in horseboxes induces a positive cognitive bias in horses. J Vet Behav. (2016) 11:50–5. 10.1016/j.jveb.2015.11.005

[30] Burdick NC Agado B White JC Matheney KJ Neuendorff DA Riley DG. Evolution of exit velocity in suckling Brahman calves. J Anim Sci. (2011) 89:233–6. 10.2527/jas.2010-2973 20852080

[31] Higashiyama Y Narita H Nashiki M Higashiyama M Kanno T. Urinary cortisol levels in Japanese shorthorn cattle before and after the start of a grazing season. Asian-Aust J Anim Sci. (2005) 18:1430–4. 10.5713/ajas.2005.1430

[32] Morrow CJ Kolver ES Verkerk GA Matthews LR. Urinary corticosteroids: an indicator of stress in dairy cattle. Proc NZ Soc Anim Prod. (2000) 60:218–21.

[33] Nakajima N Doi K Tamiya S Yayota M. Effects of grazing in a sown pasture with forestland on the health of Japanese Black cows as evaluated by multiple indicators. J Appl Anim Welf Sci. (2021) 24:173–87. 10.1080/10888705.2020.1813581 32877263

[34] Grandin T. Assessment of stress during handling and transport. J Anim Sci. (1997) 75:249–57. 10.2527/1997.751249x 9027573

[35] Burdick NC Randel RD Carroll JA Welsh TH. Interactions between temperament, stress, and immune function in cattle. Int J Zool. (2011) 2011:373197. 10.1155/2011/373197

