# Supplementary material for: Temperament Trait Changes in Japanese Black Cows Under Grazing and Confined Conditions
Source: Front Vet Sci. 2021 Sep 10;8:705764. doi: 10.3389/fvets.2021.705764 (PMC8461330; doi:10.3389/fvets.2021.705764)
Supplement: Supplementary file 1 [file Presentation_1.PPTX]

## Slide 1
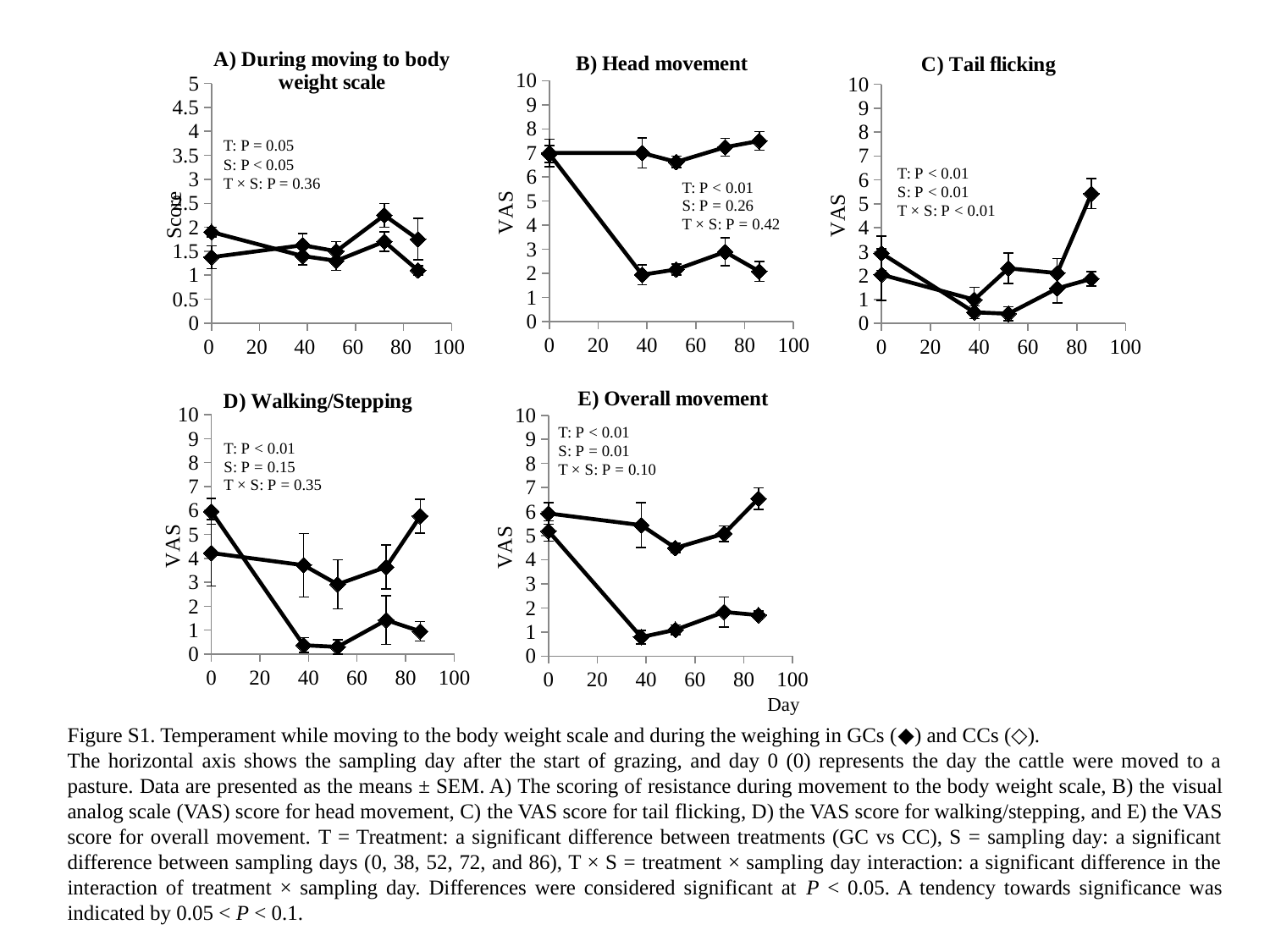

### Chart: B) Head movement
| Category | CC | GC |
|---|---|---|
### Chart: A) During moving to body weight scale
| Category | CC | GC |
|---|---|---|
### Chart: C) Tail flicking
| Category | CC | GC |
|---|---|---|
### Chart: D) Walking/Stepping
| Category | CC | GC |
|---|---|---|
### Chart: E) Overall movement
| Category | CC | GC |
|---|---|---|T: P = 0.05
S: P < 0.05
T × S: P = 0.36
Day
Figure S1. Temperament while moving to the body weight scale and during the weighing in GCs (◆) and CCs (◇).
The horizontal axis shows the sampling day after the start of grazing, and day 0 (0) represents the day the cattle were moved to a pasture. Data are presented as the means ± SEM. A) The scoring of resistance during movement to the body weight scale, B) the visual analog scale (VAS) score for head movement, C) the VAS score for tail flicking, D) the VAS score for walking/stepping, and E) the VAS score for overall movement. T = Treatment: a significant difference between treatments (GC vs CC), S = sampling day: a significant difference between sampling days (0, 38, 52, 72, and 86), T × S = treatment × sampling day interaction: a significant difference in the interaction of treatment × sampling day. Differences were considered significant at P < 0.05. A tendency towards significance was indicated by 0.05 < P < 0.1.

## Slide 2
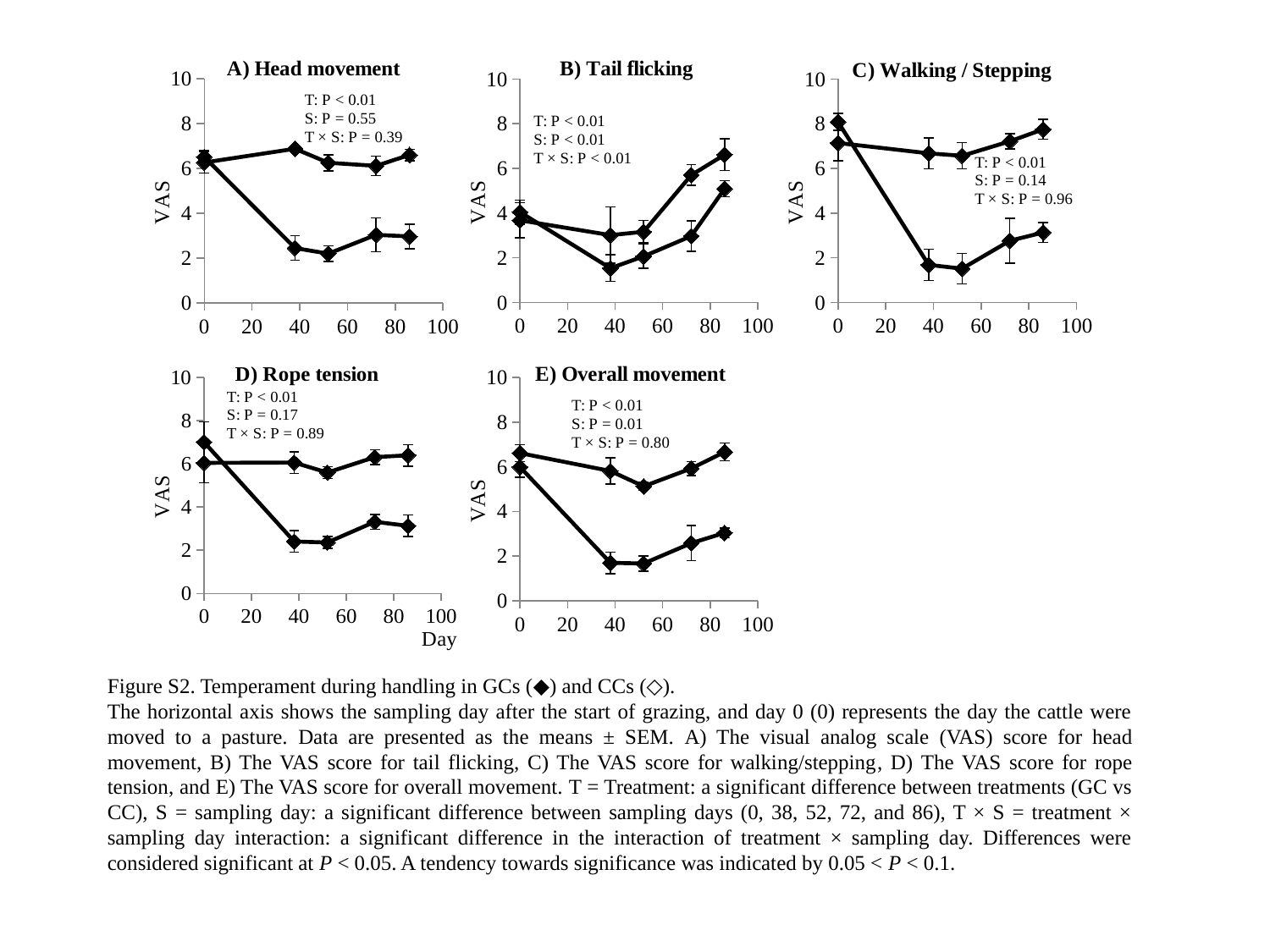

### Chart: A) Head movement
| Category | CC | GC |
|---|---|---|
### Chart: B) Tail flicking
| Category | CC | GC |
|---|---|---|
### Chart: C) Walking / Stepping
| Category | CC | GC |
|---|---|---|
### Chart: E) Overall movement
| Category | CC | GC |
|---|---|---|
### Chart: D) Rope tension
| Category | CC | GC |
|---|---|---|Figure S2. Temperament during handling in GCs (◆) and CCs (◇).
The horizontal axis shows the sampling day after the start of grazing, and day 0 (0) represents the day the cattle were moved to a pasture. Data are presented as the means ± SEM. A) The visual analog scale (VAS) score for head movement, B) The VAS score for tail flicking, C) The VAS score for walking/stepping, D) The VAS score for rope tension, and E) The VAS score for overall movement. T = Treatment: a significant difference between treatments (GC vs CC), S = sampling day: a significant difference between sampling days (0, 38, 52, 72, and 86), T × S = treatment × sampling day interaction: a significant difference in the interaction of treatment × sampling day. Differences were considered significant at P < 0.05. A tendency towards significance was indicated by 0.05 < P < 0.1.

## Slide 3
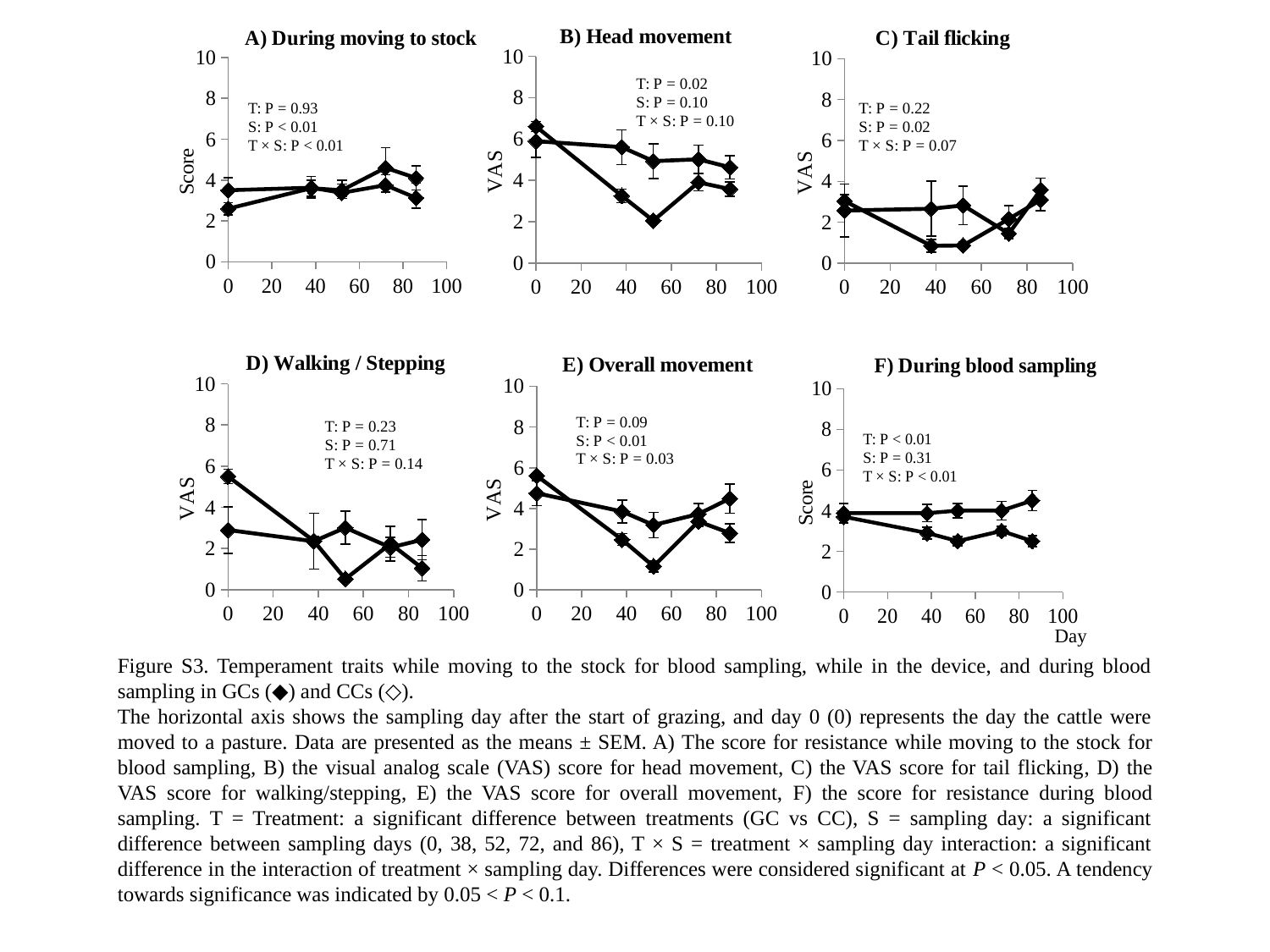

### Chart: A) During moving to stock
| Category | CC | GC |
|---|---|---|
### Chart: C) Tail flicking
| Category | CC | GC |
|---|---|---|
### Chart: B) Head movement
| Category | CC | GC |
|---|---|---|
### Chart: D) Walking / Stepping
| Category | CC | GC |
|---|---|---|
### Chart: E) Overall movement
| Category | CC | GC |
|---|---|---|
### Chart: F) During blood sampling
| Category | CC | GC |
|---|---|---|Day
Figure S3. Temperament traits while moving to the stock for blood sampling, while in the device, and during blood sampling in GCs (◆) and CCs (◇).
The horizontal axis shows the sampling day after the start of grazing, and day 0 (0) represents the day the cattle were moved to a pasture. Data are presented as the means ± SEM. A) The score for resistance while moving to the stock for blood sampling, B) the visual analog scale (VAS) score for head movement, C) the VAS score for tail flicking, D) the VAS score for walking/stepping, E) the VAS score for overall movement, F) the score for resistance during blood sampling. T = Treatment: a significant difference between treatments (GC vs CC), S = sampling day: a significant difference between sampling days (0, 38, 52, 72, and 86), T × S = treatment × sampling day interaction: a significant difference in the interaction of treatment × sampling day. Differences were considered significant at P < 0.05. A tendency towards significance was indicated by 0.05 < P < 0.1.
